# Allele-specific antibodies to *Plasmodium falciparum* merozoite surface protein-2 and protection against clinical malaria

**DOI:** 10.1111/j.1365-3024.2009.01178.x

**Published:** 2010-03

**Authors:** F H A OSIER, L M MURUNGI, G FEGAN, J TUJU, K K TETTEH, P C BULL, D J CONWAY, K MARSH

**Affiliations:** 1 KEMRI-Centre for Geographic Medicine ResearchCoast, Kilifi, Kenya; 2 Medical Research Council LaboratoriesFajara, Banjul, The Gambia; 3 London School of Hygiene and Tropical MedicineLondon, UK

**Keywords:** allele-specific antibodies, clinical malaria, *msp2* genotypes, *Plasmodium falciparum*

## Abstract

IgG and IgG3 antibodies to merozoite surface protein-2 (MSP-2) of *Plasmodium falciparum* have been associated with protection from clinical malaria in independent studies. We determined whether this protection was allele-specific by testing whether children who developed clinical malaria lacked IgG/IgG3 antibodies specific to the dominant *msp2* parasite genotypes detected during clinical episodes. We analysed pre-existing IgG and IgG1/IgG3 antibodies to antigens representing the major dimorphic types of MSP-2 by ELISA. We used quantitative real-time PCR to determine the dominant *msp2* alleles in parasites detected in clinical episodes. Over half (55%, 80/146) of infections contained both allelic types. Single or dominant IC1- and FC27-like alleles were detected in 46% and 42% of infections respectively, and both types were equally dominant in 12%. High levels of IgG/IgG3 antibodies to the FC27-like antigen were not significantly associated with a lower likelihood of clinical episodes caused by parasites bearing FC27-like compared to IC1-like alleles, and *vice versa* for IgG/IgG3 antibodies to the IC1-like antigen. These findings were supported by competition ELISAs which demonstrated the presence of IgG antibodies to allele-specific epitopes within both antigens. Thus, even for this well-studied antigen, the importance of an allele-specific component of naturally acquired protective immunity to malaria remains to be confirmed.

## Introduction

Evidence from experimentally induced malaria and epidemiological observations has been generally considered to support the idea that immunity to malaria is to an important extent ‘strain specific’ (1–3). While the concept of a malaria ‘strain’ remains contentious (4), there is evidence in animal models that the protection afforded by immunization with antigens encoded by polymorphic single locus genes is largely allele-specific (5,6). The implications are that sub-unit vaccines based on only one allelic type might protect against a fraction of the parasite population, and thus select for parasites bearing alternative alleles. This outcome was suggested in a phase IIb trial of the combination B malaria vaccine which contained the *3D7*-like allele of merozoite surface protein-2 (MSP-2), and where subsequent infections in vaccinees had a preponderance of parasites bearing the alternative *FC27*-like alleles, supporting the rationale for incorporating the major allelic types of polymorphic antigens in malaria vaccine development (7).

We have previously shown that high levels of naturally acquired IgG antibodies to MSP-2 are strongly associated with protection against clinical malaria in two independent cohorts of Kenyan children (8). Here, we investigated whether this protection is specific in relation to the major allelic dimorphisms of *msp2* in the sub-set of children who subsequently presented to hospital with malaria. We also determined whether the protective anti-MSP-2 antibodies were of the IgG1 or IgG3 sub-classes.

## Materials and methods

### Patient samples

A case–control study of clinical malaria in which antibodies to a panel of *Plasmodium falciparum* merozoite antigens including MSP-2 were analysed in children aged 1–5 years in Kilifi, Kenya has been reported in detail previously, and included 165 cases and 298 controls (8). This study focuses on the cases (*n* = 165) who presented to hospital with either mild or severe malaria, and for whom parasite DNA was available from this acute clinical episode (*N* = 146, mild malaria *n* = 71, severe malaria *n* = 75). All antibodies were assayed in serum samples collected during a cross-sectional survey at the start of a malaria transmission season in May 1995. As the antibodies were collected before the clinical episodes, they are referred to as ‘pre-existing’ antibodies. Parasite DNA was collected from subsequent samples collected when children presented to hospital with acute clinical malaria. Ethical approval was granted by the Kenya National Research Ethics Committee.

### Parasite genotyping by real-time quantitative PCR (RTQ-PCR)

#### Design of msp-2 family specific primers

Merozoite surface protein-2 sequences of *P. falciparum* contain a central domain comprised of repeats that vary in number, length and sequence, flanked in turn by nonrepetitive variable sequences, and by conserved N- and C-terminal domains. Dimorphic, nonrepetitive sequences internal to the N- and C-termini distinguish the two main allelic families (*3D7*/*IC1-like* or ‘type A’ and *FC27-like* or ‘type B’) (9). For simplicity and clarity, these two allelic families will henceforth be referred to as type A or type B. Primers were designed to amplify dimorphic regions close to the C-terminus using Primer Express Version 3·0 (Applied Biosystems, Warrington, UK), using sequences from a type A-like parasite (accession number U91677) and a type B-like parasite (accession number EU647454). The forward and reverse primers for type A alleles were MSP2ICF: 5′-CCCACTCAAGATGCAGACACTAA-3′ and MSP2ICR: 5′-TGGAGCAGAATTTTCAGCTTGTT-3′, respectively, while those for type B alleles were MSP2FCF 5′-GCACCAAATAAAACAGACGGTAAAG-3′ and MSP2FCR 5′-GGTCCTTCTTCAGTTGATTCATTTAAT-3′, respectively.

#### Real-time quantitative PCR

Reactions were performed using the 7500 Real-Time PCR System (Applied Biosystems). Twenty-five microlitre reactions were set-up in 96 well optical plates (Applied Biosytems) using Quantitect SYBR Green PCR Master Mix (Qiagen, Crawley, UK), 100 nm primers (SIGMA, Dorset, UK), 1 μL of template and the ROX™ dye for detection. Initial amplification steps were 50°C for 2 s then 95°C for 10 s, followed by 40 cycles of 95°C for 15 s and 60°C for 1 min, then a dissociation step of 95°C for 15 s and 60°C for 1 min, with a final post-dissociation cooling step of 95°C for 15 s. The dissociation step was used to exclude contamination and to determine the dissociation temperatures of the amplicons. The expected amplicon sizes were 65 and 76 base pairs for the *msp2* type A and type B alleles, respectively. The default threshold setting of 0·2 was used to analyse all the data. The average amplification efficiency was determined from the slope of the standard curve for each reaction using the equation *E* = 10^(−1/slope)^−1.

#### Preparation of genomic DNA standards for RTQ-PCR

Laboratory cultures of *P. falciparum* clones ‘Malayan Camp’ (MC, type A at the *msp2* locus) and ‘Dd2’ (type B at the *msp2* locus) were grown to 5–10% parasitaemia. A total of 100 μL of each of the culture media (20 μL pellet) was used to extract parasite genomic DNA using DNA Qiamp mini-kits (Qiagen, UK). The amount of genomic DNA was quantified by UV spectrophotometry and adjusted to 10 ng/μL for each of the two standards. Tenfold serial dilutions were then prepared for each (MC and Dd2), in the range of 10–0·00001 ng and were run separately for each allelic type on each plate to enable comparable quantification of each major allelic type.

#### Preliminary experiments with mixed clone infections

To determine the accuracy of the method for quantification of the major dimorphic types of msp2 from patient samples, mixtures of MC and Dd2 parasite cultures were prepared in the following ratios, with a final volume of 100 μL for each set: 100 : 0; 99·5 : 0·5; 99 : 1; 90 : 10; 50 : 50; 10 : 90; 99 : 1; 99·5 : 0·5; 0 : 100. A similar design has been reported previously for quantification of rodent malaria parasite genotypes (10). Parasite genomic DNA was then extracted from each of these blood mixtures as described above before analysis by RTQ-PCR including no-template negative controls and a positive control. Seryl-tRNA synthetase is conserved in *P. falciparum* (11) and was tested in parallel as the positive control using published primers, PF07_0073 (12). The reproducibility or between-run variation of the assay was checked and confirmed by testing each of the mixtures with each primer pair in multiple independent runs. The coefficient of variation (%) was calculated as the (row or average standard deviation/mean) × 100 (10).

#### Parasite genotyping of patient samples

Frozen parasite isolates were thawed and cultured for one cycle using standard techniques from the ring to the schizont stage, to bulk up parasite DNA for extraction using the DNA Qiamp mini-kits (Qiagen). Genomic DNA from each sample was amplified by RTQ-PCR in separate reactions on the same plate using primers for type A and type B alleles. Standards for each allelic family were run simultaneously on each plate for quantification. Samples were considered positive, if they contained >0·001 ng/μL of genomic DNA. This threshold was the lowest amount that could be reproducibly detected as determined from the standard curves run on each plate. Samples were considered to be dominant for one allelic type, if they contained at least double the amount of the alternative allelic type. The proportions of either allelic type of genomic DNA were calculated from the total amount of DNA detected in ng/μL from each allele-specific reaction. These experiments were designed to detect the dominant *msp2* allelic families and not the absolute number of clones.

### Recombinant antigens and ELISA

Detailed methods for expression, purification and characterization of the recombinant proteins used in this study are published. Both MSP-2 antigens were expressed in *Escherichia coli* fused to a Glutathione S-transferase (GST) sequence for purification, and each represents a major allelic type of MSP-2 (MSP-2_CH150/9 (type A) and MSP-2_Dd2 (type B)) (13), and the GST portion was also expressed alone as a control for background antibody reactivity. For simplicity and clarity, these antigens will henceforth be referred to as type A and type B antigens.

The total IgG ELISA assays were performed using a standard protocol, and results on the children with severe malaria (*n* = 89) have been reported as part of a previous analysis (8). Thresholds for characterizing IgG antibody reactivity as ‘high level’ had been derived previously as the antibody ELISA OD level above which the risk of disease was lower than the population’s average risk (OD > 0·620 and 0·712 for the MSP-2 type A and B antigens, respectively) (8). Sero-positivity for total IgG was defined as an ELISA OD value above the mean plus three standard deviations of the values for negative control sera from 40 nonmalaria exposed donors from the United Kingdom. For the type A antigen, the cut-off for sero-positivity was an ELISA OD of 0·101, with a median of 0 (range 0–0·0859). For the type B antigen, the cut-off for sero-positivity was an ELISA OD of 0·109, with a median of 0 (range 0–0·098).

Competition ELISA assays were performed on selected samples to detect total IgG antibodies directed against allele-specific as opposed to conserved epitopes (shared between alleles). Diluted sera were pre-incubated with an excess (1000 ng) of competing heterologous antigens for 5 h, before addition to each ELISA plate well coated with 50 ng of homologous antigens and subsequent performance of the ELISA in the standard method. This ensured that only IgG antibodies to allele-specific epitopes of the homologous antigen were detected, while those to conserved epitopes would have been absorbed in the pre-incubation step. As a control, an aliquot of the same serum sample was also pre-incubated with an excess (1000 ng) of homologous antigen, before addition to ELISA plates containing 50 ng of the same antigen. The average ELISA OD in the homologous competition assays was 0·156 for the type A antigen and 0·221 for the type B antigens. The median difference in ELISA OD between heterologous and homologous competition assays was 0·510 (range 0·014–1·920) for the type A antigen, and 0·386 (range −0·117 to 1·918) for the type B antigen. A difference of >0·3 ELISA OD units between the heterologous and homologous absorption assays was considered to indicate substantial allele-specific reactivity (14). IgG sub-class ELISA assays were identical to those for total IgG except horseradish peroxidase (HRP)-conjugated polyclonal sheep antibodies specific for human IgG1 and IgG3 (The Binding Site, Birmingham, UK) were used as secondary antibodies at a 1/3000 dilution.

### Statistical analysis

Logistic regression was used to examine the effect of sero-positivity (or high levels) of total IgG to each MSP-2 antigen on the odds of getting a clinical episode caused by parasites bearing the corresponding homologous parasite alleles, compared with parasites bearing the alternative heterologous alleles. Analyses were conducted first for single allele infections and then repeated including children in whom a single dominant allelic type was detected. In separate analyses, the proportions of children who were sero-positive (or had high levels of IgG antibody) to each antigen were compared amongst children in whom a single or dominant corresponding allelic type was detected.

The effects of high levels of IgG1 and IgG3 antibodies against each of the MSP-2 antigens on the risk of clinical disease were analysed using modified Poisson regression models as previously described (8). These analyses were conducted on the entire data set (cases and controls), as had been previously reported for total IgG antibodies to MSP-2 (8). The results are reported as relative risks (RR) with 95% confidence intervals (95% CI) and *P*-values.

## Results

### Parasite genotyping by RTQ-PCR

**Figure 1:**
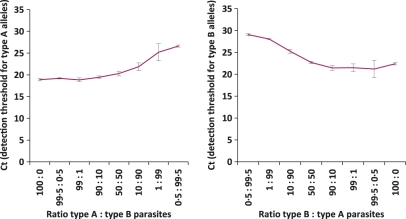
Genomic DNA extracted from laboratory cultures containing known mixtures of parasites bearing type A or type B *msp2* alleles was assayed by QRT-PCR using primers for type A and type B alleles. Left panel: as the proportion of type A bearing parasites decreased, the Ct increased proportionally. Right panel: as the proportion of type B bearing parasites increased, the Ct decreased proportionally. Error bars indicate the standard deviations for four independent runs.

The specificity of the primers for the major allelic families of *msp2* was confirmed by conducting cross-reaction tests, where type A genomic DNA (MC) was amplified with type B primers and *vice versa* [type B genomic DNA (Dd2) amplified with type A primers], with positive and negative controls. Type A genomic DNA was only amplified by type A primers and the positive control primers, but not by type B primers. Similarly, type B genomic DNA was only amplified by type B primers and the positive control primers, but not by type A primers. The dissociation curves for each allelic type showed single, allele-specific peaks free of contamination. Preliminary experiments in which laboratory cultured parasites of either *msp2* allelic type were mixed in known proportions were highly reproducible on four independent runs (Figure 1). The threshold cycle (Ct) reflects the cycle number when fluorescence generated within a reaction reaches the threshold and is inversely proportional to logarithm of the initial copy number. Figure 1 (left panel) shows that as the proportion of MC parasites in the mixture decreased the Ct increased proportionally, while Figure 1 (right panel) shows that as the proportion of Dd2 parasites in the mixture increased, the Ct decreased proportionally. The average coefficients of variation for four independent runs were 2·68% for type A assays and 0·35% for type B assays. The amplification efficiency was determined from the standard curves on each plate and was >98% for both type A and type B reactions indicating that there was no amplification bias for either allelic type.

### Proportion of isolates containing each of the major *msp2* alleles

Data on parasite genotypes was available for 146/165 study children who had a clinical episode of malaria. Parasite material was collected at the time of presentation to hospital with an acute clinical episode of malaria. One sample was negative by RTQ-PCR for both *msp2* allelic types as well as the positive control. Table 1 shows the proportions of *msp2* alleles detected by RTQ-PCR. Although type A *msp2* alleles were more common (i.e. proportion positive) than type B alleles, this was not significant (82% vs. 72%, *P* = 0·063). A single allelic type was detected in 45% of the samples, while the remainder contained both type A and type B alleles. Neither allelic type was over-represented amongst children presenting with mild or severe malaria (proportion with type A, 83% vs. 81% and type B, 70 vs. 73%, for mild and severe malaria, respectively). There was no bias in the distribution of either allele amongst all age groups of children (analysed in 1 year categories from 1 to 5 years). Dominant (including single) type A alleles were detected in 46% (67/146) of samples, while dominant type B alleles were detected in 42% (61/146) of samples. Neither allelic type was dominant in children presenting with mild or severe malaria, or in any particular age category. A dominant allelic type (defined as at least double the amount of the alternative allelic type) could not be confidently distinguished in a minority of samples, 18/146 (12%).

**Table 1:** Number of isolates containing each of the major *msp2* alleles

	Type B		
Type A	0	1	Total
0	1	25	26
1	40	80	120
Total	41	105	146

### Prevalence of total IgG antibodies to MSP-2

**Figure 2:**
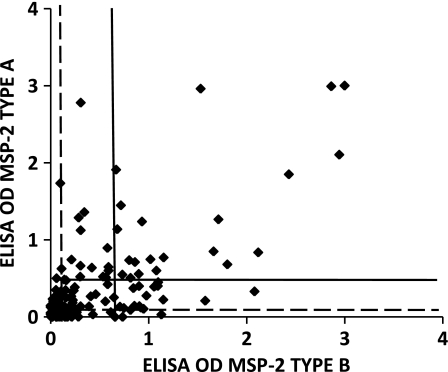
Correlation between IgG antibody reactivity to MSP-2 type A and B antigens. Dashed lines indicate cut-off values for sero-positivity, while solid lines indicate cut-off values for high levels of antibodies. Pairwise correlation coefficient was 0·67, *P* < 0·001 (*n* = 146).

Antibodies were analysed in samples collected during a cross-sectional survey at the start of a malaria transmission season. This was before the children presented with acute clinical episodes. Overall, the IgG antibody prevalence and levels to both MSP-2 antigens were similar and reflected the prevalence of the *msp2* allelic types (above). The prevalence of IgG sero-positivity was 65% (95/146) for the type A antigen and 65% (95/146) for the type B antigen, while that of high level IgG antibodies was 20% (29/146) and 25% (37/146) for the type A and B antigens, respectively. Total IgG antibodies to the type A and B antigens were highly correlated, pairwise correlation coefficient *P* = 0·67, *P* < 0·001, and only a minority of children showed clear allele-specific IgG antibody reactivity when antibodies to full-length MSP-2 antigens were examined by indirect ELISA (Figure 2). Total IgG antibody levels to the MSP-2 type A and B antigens increased significantly with age (*P* = 0·020 and *P* = 0·004, respectively).

### IgG3 antibodies to MSP-2 are associated with protection from clinical malaria

We had previously reported that total IgG antibodies to MSP-2 were strongly associated with protection from clinical malaria in this population (8). We further analysed these samples to determine whether this protection was mediated by antibodies of the IgG1 or IgG3 sub-classes. For the MSP-2 type A antigen, IgG3 sub-class antibodies predominated over IgG1 antibodies (Wilcoxon matched-pairs sign-rank test *z* = −7·3, *P* < 0·001), while for the MSP-2 type B antigen the distributions of IgG1 and Ig3 antibodies were similar (Wilcoxon matched-pairs sign-rank test *z* = 0·5, *P* = 0·587). We found that IgG3 antibodies to both the type A and B antigens were significantly associated with protection from clinical malaria, while IgG1 antibodies to both antigens were not. Children with high levels of IgG3 to the MSP-2 type A and B antigens were significantly less likely to develop clinical disease than those with low levels, RR 0·35 (95% CI 0·14–0·88) *P* = 0·026, and RR 0·22 (95% CI 0·08–0·60) *P* = 0·003, respectively. In contrast, children with high levels of IgG1 to the type A antigen were not protected from clinical malaria when compared to those with low levels (RR 1·04 (95% CI 0·56–1·93) *P* = 0·885). Although children with high levels of IgG1 to the type B antigen were less likely to develop clinical malaria than those with low levels, this was not significant [RR 0·57 (95% CI 0·30–1·12) *P* = 0·108].

### Total IgG, IgG1 and IgG3 antibodies to the major allelic MSP-2 antigens do not appear to protect in an allele-specific manner

If antibodies conferred protection in an allele-specific manner, it might be expected that antibodies to the type A MSP-2 antigen would protect against clinical episodes caused by parasites bearing homologous type A as opposed to type B *msp2* alleles. We therefore investigated whether children sero-positive for type A IgG antibodies were less likely to be infected with parasites containing type A compared to type B alleles, than sero-negative children and *vice versa* for type B IgG antibodies and alleles (Table 2). To account for the fact that children living in malaria-endemic areas commonly harbour parasites asymptomatically and therefore parasites detected may not be causally responsible for clinical episodes, we conducted two separate analyses. The first considered only those in whom a single allelic type was detected, while the second additionally included those in whom a dominant allelic type was detected. As previous analyses in this cohort of children demonstrated that defining a threshold for high vs. low level IgG antibodies allowed a robust prediction of children who were subsequently protected from clinical malaria (8), we repeated the above analyses using this threshold in place of sero-positivity.

**Table 2:** Logistic regression for the effect of type A (or B) MSP-2 IgG antibodies on clinical episodes caused by parasites bearinghomologous vs. heterologous alleles

	Single allele infections		Single and dominant allele infections	
Antibodies	Odds ratio 95% CI	*P*-value	Odds ratio 95% CI	*P*-value
Sero-positive A	0·84 (0·30–2·37)	0·747	1·07 (0·52–2·23)	0·837
High level A	2·88 (0·55–14·81)	0·207	1·96 (0·80–4·81)	0·140
Sero-positive B	0·76 (0·27–2·11)	0·603	1·20 (0·58–2·48)	0·608
High level B	0·44 (0·12–1·57)	0·209	0·53 (0·23–1·21)	0·134

For type A IgG antibodies, the odds ratios indicate the protective effects of sero-positivity for (or high levels of) type A IgG antibodies on clinical episodes with parasites bearing type A as opposed to B alleles. Similarly, for type B IgG antibodies, the odds ratios indicate the protective effects of sero-positivity for (or high levels of) type B IgG antibodies on clinical episodes with parasites bearing type B as opposed to A alleles. Single allele infections, *n* = 65 (40 type A and 25 type B alleles, respectively). Single and dominant clone infections, *n* = 128 (67 type A and 61 type B alleles, respectively).

Pre-existing type A IgG antibodies did not protect against clinical episodes caused by parasites bearing type A as opposed to type B *msp2* alleles. This was true both for single allele infections and infections in which dominant alleles were additionally considered (Table 2). Children with pre-existing type B IgG antibodies were not significantly less likely to present with clinical episodes caused by parasites bearing type B as opposed to type A alleles (Table 2). These results did not change when the data were stratified for severe or mild malaria. When the same data were analysed using IgG1 and IgG3 antibodies, there was also no evidence that either of these IgG sub-classes protected against clinical episodes with parasites bearing homologous as opposed to heterologous alleles.

In a separate analysis, we considered whether children presenting with single or dominant (single plus dominant) type A infections were often sero-negative for the corresponding type A IgG antibodies, and *vice versa* for type B. This relationship was not observed (Table 3, top panel). Similarly, children presenting with type A alleles had low levels of pre-existing type A IgG antibodies, but also had low levels of pre-existing type B antibodies. Likewise, children presenting with type B alleles had low levels of pre-existing type B IgG antibodies, but also had low levels of pre-existing type A IgG antibodies. These findings indicate that the lack of protection was not allele-specific (Table 3, bottom panel). Similar results were obtained, when the data were stratified for severe or mild malaria. When these data were analysed using IgG1 and IgG3 sub-class antibodies, children presenting with either type A or type B infections were often sero-negative or had low levels of antibodies to both antigens, indicating that the lack of protection was not allele-specific.

**Table 3:** Prevalence of sero-positivity or high level IgG antibodies for MSP-2 antigens amongst children with a single or dominant *msp2* major allelic type

	MSP-2 antibodies			
*msp2* allelic type	Sero-positive A *n* (%)	Sero-negative A *n* (%)	Sero-positive B *n* (%)	Sero-negative B *n* (%)
Single type A alleles (*n* = 40)	24 (60)	16 (40)	25 (62·5)	15 (37·5)
Single type B alleles (*n* = 25)	16 (64)	9 (36)	14 (56)	11 (44)
Dominant type A alleles (*n* = 67)	44 (66)	23 (34)	41 (61)	26 (39)
Dominant type B alleles (*n* = 61)	39 (64)	22 (36)	40 (66)	21 (34)

*msp2* allelic type	High level A *n* (%)	Low level A *n* (%)	High level B *n* (%)	Low level B *n* (%)

Single type A alleles (*n* = 40)	8 (20)	32 (80)	12 (30)	28 (70)
Single type B alleles (*n* = 25)	4 (16)	21 (84)	2 (8)	23 (92)
Dominant type A alleles (*n* = 67)	17 (25)	50 (75)	21 (31)	46 (69)
Dominant type B alleles (*n* = 61)	9 (15)	52 (85)	12 (20)	49 (80)

Similar proportions of children with single or single plus dominant allele type A or B infections were sero-positive for pre-existing type A and type B IgG antibodies, respectively. Children infected with type A or B alleles had low levels of IgG antibodies to the homologous antigen, but also had similarly low levels of IgG antibodies to the heterologous antigen, indicating that the lack of protection was not allele-specific.

### Competition ELISA confirms the presence of antibodies to allele-specific epitopes

It may be argued that because full-length antigens utilized in this study contain allele-specific as well as conserved epitopes, the contribution of allele-specific epitopes *per se* to protection cannot be directly ascertained. To address this, we selected children whose clinical episodes were caused by parasites containing a dominant (or single) *msp2* allelic type and who had high levels of pre-existing IgG antibodies to the homologous MSP-2 antigen. We performed competition ELISAs on these sera to verify that they contained IgG antibodies to allele-specific epitopes within the MSP-2 antigens. Sixteen children had high levels of pre-existing IgG antibodies to MSP-2 type A and subsequent clinical episodes caused by type A parasites. Competition with excess of the type B antigen revealed that 13/16 children had IgG antibodies to allele-specific epitopes within the type A antigen. Eleven children had high levels of pre-existing IgG antibodies to the type B antigen and subsequent clinical episodes caused by type B parasites. Competition with excess of the type A antigen revealed that 8/11 children had IgG antibodies to allele-specific epitopes within the type B antigen. Thus, the majority of these children developed clinical episodes despite having allele-specific IgG antibodies homologous to the major allelic types of the parasites that infected them.

## Discussion

Studies conducted in different populations have shown that serum IgG or IgG3 antibodies to the blood stage antigen MSP-2 of *P. falciparum* were associated with protection from clinical episodes of malaria (8,13,15). This antigen is encoded by a polymorphic single locus gene and there is strong evidence that antigenic diversity has been driven by immune pressure (16). Along with this, the combination B malaria vaccine trial provided the first proof-of-principle of vaccine-induced selection on malaria parasites in humans (7).

Children and adults in malaria-endemic areas commonly harbour asymptomatic parasitaemias, comprising complex mixtures of distinct parasite clones, which fluctuate over months, weeks, and even days (3,17). These longitudinal studies have also shown that clinical episodes are often associated with an abrupt increase in parasite densities (1) in which novel parasite clones are commonly detected (1,18,19), consistent with the idea of ‘strain’-specific immunity, limiting the growth of some, but not all parasites (20). We therefore used quantitative real-time PCR to determine the dominant parasite genotype detectable (10), with the cautious assumption that it was causally responsible for the clinical episode of malaria.

In the population studied here, we previously found that high levels of IgG antibodies to MSP-2 were strongly associated with protection against clinical malaria (8). Further analyses revealed that this protection was mediated by antibodies of the IgG3 and not the IgG1 sub-class. In this study, we analysed the sub-group of children who subsequently developed clinical episodes to determine whether antibodies conferred protection in an allele-specific manner. In this sub-group of children, neither pre-existing total IgG, nor IgG1/IgG3 antibodies differentially protected against parasites bearing homologous as opposed to heterologous *msp2* alleles. For total IgG antibodies, the presence of antibodies to allele-specific epitopes was confirmed by competition ELISAs, and supported the finding that protection was not mediated in an allele-specific fashion. These findings concur with those recently reported from Papua New Guinea where although IgG1 and IgG3 antibodies to both type A and B antigens were associated with protection, it was not allele-specific. While the majority of infections were caused by parasites bearing type A alleles, high levels of IgG1 and IgG3 antibodies to the type B antigen (as well as the type A antigen) protected against symptomatic episodes caused by type A parasites (21).

While many studies have examined the infecting MSP-2 parasite genotype and the corresponding allele-specific antibody response, these have been performed in concurrent samples (22–24) or in acute-convalescent pairs of samples (25,26). To our knowledge only one study has analysed naturally acquired allele-specific IgG antibodies to MSP-2 and protection from clinical malaria, albeit using a slightly different end-point (27). This study was conducted amongst adults in the Brazilian Amazon who were exposed to low levels of *P. falciparum* transmission. Although the sample size was limited, they found that pre-existing allele-specific IgG antibodies did not predict clinical outcome in infections with parasites bearing homologous rather than heterologous *msp2* alleles. Clinical outcome was defined as the prevalence or severity of symptomatic infections, though none of their study participants developed severe malaria during follow-up (27). A limitation of our study is that we did not assay the fine levels of allele-specificity to the many variants or sub-types of the major allelic families of MSP-2 (28). Rather, we concentrated on the concern that vaccine-induced immunity may be limited by specificity at the level of major allelic types. We found little evidence that this was the case for naturally acquired protective responses in this population. Another potential limitation is that our competition ELISAs were performed with total IgG antibodies, rather than with the IgG3 sub-class antibodies which were specifically associated with protection. On the other hand, we found no evidence that pre-existing IgG3 antibodies conferred protection in an allele-specific manner in this sub-group of children.

Taken together, these data show that although pre-existing IgG/IgG3 antibodies to antigens representing the major dimorphic types of MSP-2 are strongly associated with a reduced incidence of clinical malaria in the majority of children (8), a minority still develop disease despite having had not only high level antibodies, but also antibodies to allele-specific epitopes within those antigens. This minority of children may not be the ideal group to study as they represent the ‘failures’, in whom putatively ‘protective’ anti-MSP-2 antibodies failed to protect. Another potential explanation for this result is the time to presentation to hospital with an acute clinical episode which varied widely in our study, mean 139 days (range 7–274). Given the half life of IgG antibodies to several malaria antigens including MSP-2 may be short in young children in endemic areas (29,30), it is likely that the levels of antibodies had declined significantly by the time the clinical episode developed. However, one would expect that memory B cells of the corresponding specificity would remain. All the same, antibodies measured at a single time point have been a robust predictor of the risk of clinical malaria in numerous immuno-epidemiological studies, including the cohort from which the children in this study were drawn (8). Alternatively, the antibodies were present but lacked the functional capacity to control the infection and subsequent clinical presentation. Another possibility is that the intrinsically unstructured nature of MSP-2 makes it difficult to accurately detect antibody reactivity to various parts of the protein using ELISA techniques with GST fusion proteins (31).

These data should be considered in the context of the evidence for immune selection on parasite diversity (16) and the paradigm of ‘strain’ specific immunity being important in protection from malaria. While the loci used to detect the presence of distinct parasite clones or ‘strains’, are often identical to those that encode for antigens that are considered as targets of protective immunity (e.g. MSP-1 block 2, MSP-2 and MSP-3), ‘strain’-specific immunity is not synonymous with allele-specific immunity, the latter at best being a ‘sub-set’ of the former. Nonetheless, when protective antibody responses are directed to polymorphic antigens, it might be expected that the polymorphism is driven by immune pressure and that protection would be greater against disease episodes caused by parasites bearing homologous alleles. It is possible that the differential allele-specific protection necessary to maintain diversity in the population need only be marginal and could not be detected in this kind of study. Alternatively, signatures of selection on polymorphic parts of antigens may identify them as targets of protective immunity, but do not necessarily indicate that they are more important targets than adjacent sequences in the same proteins that may be conserved for functional reasons. Protective immunity might be directed against conserved sequences within antigenically diverse proteins. This would be welcome news for malaria vaccine development and needs to be confirmed in larger studies, as well as in other malaria transmission settings.

## References

[1] Contamin H, Fandeur T, Rogier C (1996). Different genetic characteristics of *Plasmodium falciparum* isolates collected during successive clinical malaria episodes in Senegalese children. Am J Trop Med Hyg.

[2] Covell G, Nicol WD (1951). Clinical, chemotherapeutic and immunological studies on induced malaria. Br Med Bull.

[3] Daubersies P, Sallenave-Sales S, Magne S (1996). Rapid turnover of *Plasmodium falciparum* populations in asymptomatic individuals living in a high transmission area. Am J Trop Med Hyg.

[4] McKenzie FE, Smith DL, O’Meara WP, Riley EM (2008). Chapter 1 strain theory of malaria the first 50 years. Adv Parasitol.

[5] Crewther PE, Matthew ML, Flegg RH, Anders RF (1996). Protective immune responses to apical membrane antigen 1 of *Plasmodium chabaudi* involve recognition of strain-specific epitopes. Infect Immun.

[6] Renia L, Ling IT, Marussig M (1997). Immunization with a recombinant C-terminal fragment of *Plasmodium yoelii* merozoite surface protein 1 protects mice against homologous but not heterologous *P. yoelii* sporozoite challenge. Infect Immun.

[7] Genton B, Betuela I, Felger I (2002). A recombinant blood-stage malaria vaccine reduces *Plasmodium falciparum* density and exerts selective pressure on parasite populations in a phase 1-2b trial in Papua New Guinea. J Infect Dis.

[8] Osier FH, Fegan G, Polley SD (2008). Breadth and magnitude of antibody responses to multiple *Plasmodium falciparum* merozoite antigens are associated with protection from clinical malaria. Infect Immun.

[9] Felger I, Steiger S, Hatz C, Smith T, Beck HP (2003). Antigenic cross-reactivity between different alleles of the *Plasmodium falciparum* merozoite surface protein 2. Parasite Immunol.

[10] Cheesman SJ, de Roode JC, Read AF, Carter R (2003). Real-time quantitative PCR for analysis of genetically mixed infections of malaria parasites: technique validation and applications. Mol Biochem Parasitol.

[11] Bozdech Z, Llinas M, Pulliam BL (2003). The transcriptome of the intraerythrocytic developmental cycle of *Plasmodium falciparum*. PLoS Biol.

[12] Salanti A, Staalsoe T, Lavstsen T (2003). Selective upregulation of a single distinctly structured var gene in chondroitin sulphate A-adhering *Plasmodium falciparum* involved in pregnancy-associated malaria. Mol Microbiol.

[13] Taylor RR, Allen SJ, Greenwood BM, Riley EM (1998). IgG3 antibodies to *Plasmodium falciparum* merozoite surface protein 2 (MSP2): increasing prevalence with age and association with clinical immunity to malaria. Am J Trop Med Hyg.

[14] Osier FH, Polley SD, Mwangi T (2007). Naturally acquired antibodies to polymorphic and conserved epitopes of *Plasmodium falciparum* merozoite surface protein 3. Parasite Immunol.

[15] Metzger WG, Okenu DM, Cavanagh DR (2003). Serum IgG3 to the *Plasmodium falciparum* merozoite surface protein 2 is strongly associated with a reduced prospective risk of malaria. Parasite Immunol.

[16] Conway DJ, Polley SD (2002). Measuring immune selection. Parasitology.

[17] Farnert A, Snounou G, Rooth I, Bjorkman A (1997). Daily dynamics of *Plasmodium falciparum* subpopulations in asymptomatic children in a holoendemic area. Am J Trop Med Hyg.

[18] Magesa SM, Mdira KY, Babiker HA (2002). Diversity of *Plasmodium falciparum* clones infecting children living in a holoendemic area in north-eastern Tanzania. Acta Trop.

[19] Ofosu-Okyere A, Mackinnon MJ, Sowa MP (2001). Novel *Plasmodium falciparum* clones and rising clone multiplicities are associated with the increase in malaria morbidity in Ghanaian children during the transition into the high transmission season. Parasitology.

[20] Lines J, Armstrong JR (1992). For a few parasites more: inoculum size, vector control and strain-specific immunity to malaria. Parasitol Today.

[21] Stanisic DI, Richards JS, McCallum FJ (2009). Immunoglobulin G subclass-specific responses against *Plasmodium falciparum* merozoite antigens are associated with control of parasitemia and protection from symptomatic illness. Infect Immun.

[22] Kanunfre KA, Leoratti FM, Hoffmann EH (2003). Differential recognition of *Plasmodium falciparum* merozoite surface protein 2 variants by antibodies from malaria patients in Brazil. Clin Diagn Lab Immunol.

[23] Polley SD, Conway DJ, Cavanagh DR (2006). High levels of serum antibodies to merozoite surface protein 2 of *Plasmodium falciparum* are associated with reduced risk of clinical malaria in coastal Kenya. Vaccine.

[24] Tami A, Grundmann H, Sutherland C (2002). Restricted genetic and antigenic diversity of *Plasmodium falciparum* under mesoendemic transmission in the Venezuelan Amazon. Parasitology.

[25] Ekala MT, Jouin H, Lekoulou F, Mercereau-Puijalon O, Ntoumi F (2002). Allelic family specific humoral responses to merozoite surface protein 2 (MSP2) in Gabonese residents with *Plasmodium falciparum* infections. Clin Exp Immunol.

[26] Weisman S, Wang L, Billman-Jacobe H (2001). Antibody responses to infections with strains of *Plasmodium falciparum* expressing diverse forms of merozoite surface protein 2. Infect Immun.

[27] Scopel KK, da Silva-Nunes M, Malafronte RS, Braga EM, Ferreira MU (2007). Variant-specific antibodies to merozoite surface protein 2 and clinical expression of *Plasmodium falciparum* malaria in rural Amazonians. Am J Trop Med Hyg.

[28] Franks S, Baton L, Tetteh K (2003). Genetic diversity and antigenic polymorphism in *Plasmodium falciparum*: extensive serological cross-reactivity between allelic variants of merozoite surface protein 2. Infect Immun.

[29] Akpogheneta OJ, Duah NO, Tetteh KK (2008). Duration of naturally acquired antibody responses to blood-stage *Plasmodium falciparum* is age dependent and antigen specific. Infect Immun.

[30] Kinyanjui SM, Conway DJ, Lanar DE, Marsh K (2007). IgG antibody responses to *Plasmodium falciparum* merozoite antigens in Kenyan children have a short half-life. Malar J.

[31] Low A, Chandrashekaran IR, Adda CG (2007). Merozoite surface protein 2 of *Plasmodium falciparum*: expression, structure, dynamics, and fibril formation of the conserved N-terminal domain. Biopolymers.

